# Magnitude and factors associated with anxiety and depression among patients with breast cancer in central Ethiopia: A cross-sectional study

**DOI:** 10.3389/fpsyt.2022.957592

**Published:** 2022-08-26

**Authors:** Winini Belay, Wajana Lako Labisso, Wondemagegnehu Tigeneh, Mirgissa Kaba, Werissaw Haileselassie

**Affiliations:** ^1^Department of Reproductive Health and Health Service Management, School of Public Health, Addis Ababa University, Addis Ababa, Ethiopia; ^2^Department of Pathology, School of Medicine, College of Health Sciences, Addis Ababa University, Addis Ababa, Ethiopia; ^3^Department of Oncology, School of Medicine, College of Health Sciences, Addis Ababa University, Addis Ababa, Ethiopia; ^4^Department of Preventive Medicine, School of Public Health, Addis Ababa University, Addis Ababa, Ethiopia

**Keywords:** anxiety, depression, breast cancer, mental health, public health, Ethiopia

## Abstract

**Background:**

Patients with breast cancer are assumed to be susceptible to anxiety and depression, while there is a lack of evidence about the epidemiology and underlying factors in Ethiopia. Hence, we aimed to assess the prevalence and predictors of anxiety and depression among patients with breast cancer in Ethiopia.

**Method:**

An institutional-based cross-sectional study was conducted among patients with breast cancer who were attending treatment at the Oncology Center of the Tikur Anbessa Specialized Hospital, the largest referral teaching hospital in Ethiopia. The Hospital Anxiety and Depression Scale (HDAS) was used to evaluate the anxiety and depression status of the participants, and a structured questionnaire was used to collect sociodemographic, socioeconomic, and clinical data from the participants. Medical records of the participants were reviewed to collect and correlate information about the grade and stage of cancer. An analysis was done using STATA 15.1.

**Results:**

The study included 333 randomly selected participants, of which 35.4% were on stage III and 33% on stage II, and 67.9% were on chemotherapy and surgery. The prevalence of anxiety and depression were 60.7 and 58.6%, respectively. Poor patient-provider interaction (adjusted odds ratio [AOR] = 4.5, 95% CI: 2.49, 8.12) and having no financial support (AOR = 2.83, 95% CI: 1.63, 4.91) were the significant predictors of anxiety. Age >49 years (AOR = 0.48, 95% CI: 0.25, 0.90), poor patient-provider interactions (AOR = 3.36, 95% CI: 1.87, 6.02), and having no financial support (AOR = 3.36, 95% CI: 1.95, 5.79) were the significant predictors of depression.

**Conclusion:**

In this study, the prevalence of anxiety and depression was high among patients with breast cancer, and the lack of financial support and poor patient-provider interactions were significant predictors of anxiety and depression among these groups of patients. Patients with cancer need regular screening for mental health and better emotional support from their healthcare providers and families to prevent and treat anxiety and depression.

## Introduction

According to the GLOBOCAN (Global Cancer Statistics) 2020 report, breast cancer is one of the most commonly diagnosed malignancies and the fifth leading cause of cancer-related deaths, with an estimated 2.3 million new cases worldwide (1).

In Africa, breast cancer caused an estimated 74,072 deaths with 168,690 cases estimated to have occurred in 2018 (2). In addition, in Sub-Saharan Africa, the number of breast cancer cases was 22.4 per 100,000 in that same year (3). In Ethiopia, annual incidence and mortality due to breast cancer were reported to be 19 and 12 per 100,000 total population, respectively (4).

The proportion of the global population with depression was estimated to be 4.4% (5) while anxiety was 7.3% (6). For several factors, patients with cancer are vulnerable to stress, such as diagnostic stress, exhausting treatments, and persistent pain. Anxiety can impair medication compliance and adversely affect prognosis as well as survival rates (7). Therefore, the importance of the psychological state of patients is an integral aspect of oncological care (8).

The diagnosis of breast cancer may be devastating and can cause many adverse reactions for most women, causing signs of psychological distress, such as anxiety, depression, fatigue, discomfort, difficulty focusing, social alienation, sexuality concerns, and self-blame (9, 10). The most common co-morbid medical conditions in patients with cancer are depression and anxiety, with between 62.5 and 85% of patients with cancer meeting the criteria for depression and anxiety disorders (9, 11).

Depression is highest during the acute phase of cancer treatment, with feelings of hopelessness as part of the syndrome of depression that may influence the refusal of cancer treatment (12). Anxiety is described as an uncomfortable subjective reaction to the threat (13).

The global prevalence of depression among patients with breast cancer was 32.2% (14), while the prevalence of anxiety and depression among patients with cancer in low-and middle-income countries was 21% for major depression and 18% for anxiety disorders (15).

Patients often feel anxiety before treatment, while depression only happens after treatment (16, 17). This indicates that diagnosis is marked by anxiety, while depression is more common after treatment. Anxiety also correlates more with future concerns, such as treatment outcomes (18).

A study conducted in Addis Ababa, Ethiopia reported that the prevalence of depression among patients with breast cancer was 25% (19) and another study done in Southern Ethiopia reported the prevalence of depression and anxiety among patients with different types of cancer was 58.8 and 60%, respectively (20). In Ethiopia, many issues related to breast cancer are still uncovered. The psychiatric squeal is one of them. As a result, there is a need to investigate the burden of psychiatric suffering among patients with breast cancer. Moreover, the factors influencing the occurrence of the problems are yet to be assessed. Hence, the current study is designed to assess the magnitude and factors associated with anxiety and depression among patients with breast cancer in central Ethiopia.

## Methods

### Study area and design

The study was conducted at the Tikur Anbessa Specialized Teaching Hospital (TASH) Oncology Center, Addis Ababa, Ethiopia. TASH is the only radiotherapy center for more than 110 million people in the country. The hospital gives service to the population in Addis Ababa and peripheral parts of the country by receiving referrals from all over the country (21).

We conducted an institutional-based cross-sectional study at the TASH Oncology Center from March to September 2019.

### Study participants

The study participants were patients with breast cancer aged 18 years and older visiting TASH, Addis Ababa, Ethiopia, during the mentioned study period. Patients who had severe physical illness (those that were physically in pain during the interview) and severe mental illness (psychosis and mania) were excluded from the study.

### Sample size

The sample size was estimated with a single population proportion based on the prevalence of anxiety and depression being 31.7 and 22%, respectively, among patients with breast cancer. With a 10% non-response rate, an assumption of a 95% level of confidence, the design effect 1, and a 5% margin of error, the final sample size was 366 (22).

### Instrument and data collection procedures

The Ethiopian validated Hospital Anxiety and Depression Scale (HADS) was used to assess depression and anxiety (23), which demonstrated good consistency between the items and high test-retest reliability (24). HADS is a widely used instrument for measuring psychological distress in non-psychiatric patients (11, 25, 26). The items are rated on a four-point Likert scale ranging from 0 to 3, giving maximum and minimum scores of 0 and 21, respectively for each subscale. Sub-scores on the anxiety or depression sub-scales ranging from 0 to 7 are considered normal; while 8 to 10 and 11 to 21 are considered ‘cause for concern’ and ‘probable cases of anxiety or depression,’ respectively (24).

The interpretation of the HADS is as follows:-

(1) 0–7 is normal, (2) 8–10 is mild, (3) 11–14 is moderate, and (4) 15–21 is severe.

Additionally, patients were directly interviewed using a standard questionnaire adopted from the Ethiopian demographic and health survey (EDHS) consisting of the socio-demographic and economic characteristics, such as sex, age, educational status, marital status, and socio-economic status (27), whereas clinical characteristics of patients, such as the disease condition of the patient, were acquired directly by asking patients. Information related to tumor size, grade, and stage of cancer as well as metastasis was obtained using the medical records of the patients. The quintile (socio-economic) status of the participants was calculated using the principal component analysis (PCA). The PCA has five classifications and for the cell values to fit the multiple logistic regression model; it is re-categorized into three. Concerning the patient-provider and patient-family communication, those participants who discussed the following (treatment type, duration, all their worries, medical expenses, and their fear concerning the disease) are classified as having good communication, whereas those who discussed only few items as treatment type and duration were classified as having poor communication. Emotional support was assessed by asking the patients whether they have someone to share their feelings with in times of sadness, anger, or feeling down. Financial support was also assessed by directly asking the participants whether they have financial support or not.

### Data management and analysis

Following the data collection, the questionnaire's consistency and completeness were thoroughly examined. Both the questionnaire and the variables were coded, and the primary investigator also verified for missing values and outliers. Then, the data were entered and cleaned using Epi DATA version 4.4.2.1. Descriptive statistical techniques were employed to summarize socio-demographic and clinical data. STATA 15.1 was used for the analysis. A bivariate analysis was used to determine the unadjusted correlations between sociodemographic, clinical variables, and depression as well as anxiety. Using a multiple logistic regression model that took into account variables that were significant in the bivariate analysis, the modified effects of various variables on the likelihood of developing depression and anxiety were examined.

## Results

### Baseline characteristics of patients with breast cancer at TASH

A total of 366 patients were approached for the completion of the questionnaire; however, only 333 were included in the study, giving a response rate of 91%. In total, 20 patients refused to participate in the study, whereas 13 were unable to complete the interview due to their current conditions.

Socio-demographic, economic, financial and emotional support, and communication-related characteristics of study participants are indicated below (Table 1). The majority of the study participants were female patients with breast cancer (96%).

**Table 1:** The socio-demographic, economic, financial support, emotional support, and communication-related characteristics of patients with breast cancer.

**General characteristics**	**Frequency**	**Percent**
Gender		
Female	320	96.10%
Male	13	3.90%
Age		
19–35	93	27.93%
36–48	139	41.74%
>49	101	30.33 %
Wealth quintile		
Poor	178	53.45%
Middle	55	16.52%
Rich	100	30.03%
Religion		
Orthodox	243	72.97%
Catholic	7	2.10%
Protestant	45	13.51%
Muslim	33	9.91%
Other	5	1.50%
Education		
No formal Education	76	22.82 %
Primary school	61	18.32%
Secondary school	117	35.14%
Technical school and above	79	23.72%
Marital Status		
Single	49	14.71%
Married	169	50.75%
Other	115	34.53%
Exercise		
Yes	91	27.33 %
No	242	72.67%
Financial support		
Yes	191	57.36%
No	142	42.64%
Enough support		
Yes	78	40.83%
No	113	59.16%
Emotional support		
Yes	303	91.27%
No	30	9.01%
Patient-family communication		
Good	258	77.48 %
Poor	75	22.52%
Patient-provider communication		
Good	205	61.56%
Poor	128	38.44%

### Clinical characteristics of patients with breast cancer

More than half of the study participants were at advanced stages (stages 2–4) of breast cancer, whereas nearly 5% of them were stage 1. The majority of the study participants (226, 67.87%) had mastectomy followed by chemotherapy. The details of clinical characteristics are described in Table 2.

**Table 2:** Clinical characteristics of patients with breast cancer.

**General characteristics**	**Frequency**	**Percent**
Cancer stage		
Stage 1	17	5.11
Stage 2	110	33.03
Stage 3	118	35.44
Stage 4	88	26.43
Metastasis		
Yes	54	16.22
No	279	83.78
Treatment kind		
Chemotherapy	31	9.31
Radiotherapy	3	0.90
Chemotherapy and radiotherapy	5	1.50
Chemotherapy and surgery	226	67.87
Chemotherapy, radiotherapy, and surgery	33	9.91
Radiotherapy and surgery	3	0.90
Hormonal therapy	32	9.61
Other disease condition		
Yes	49	14.71
No	284	85.29

### Prevalence of anxiety and depression among patients with breast cancer

The prevalence of anxiety and depression was found to be 60.66 and 58.56%, respectively. The majority of patients with breast cancer met the HADS criteria for anxiety and depression.

### Factors affecting anxiety and depression among patients with breast cancer

The bivariate analysis was done to select significant predictors. Those predictors that are statistically significant in bivariate analysis were entered into multiple logistic regression to control the effect of confounding variables. The result of multiple logistic regression showed that poor patient-provider communication, having no financial support, and being in the middle and rich quintile status were statistically significant for anxiety. In addition, being age >49 years, having no financial support, poor patient-provider communication, and being in the middle quintile status were found to be significant for depression.

Compared with those having good patient-provider communication, the odds of developing anxiety were 4.5 times higher among those having poor patient-provider communication (adjusted odds ratio [AOR] = 4.5, 95% CI: 2.49, 8.12). Compared with patients who had financial support, the odds of developing anxiety were 2.83 times greater among those who had no financial support (AOR = 2.83, 95% CI: 1.63, 4.91). Compared with patients in the poor quintile range, the odds of developing anxiety were 2.86 times higher among patients in the middle quintile range (AOR = 2.86, 95% CI: 1.33, 6.17) and 2.84 times higher among patients in the rich quintile range (AOR = 2.84, 95% CI: 1.46, 5.46) (Tables 3, 4).

**Table 3:** The result of logistic regression for anxiety after controlling the effect of confounding factors among patients with breast cancer.

**Characteristics**	**Anxiety**		**COR**	**AOR**
	**No**	**Yes**	**(95%CI)**	**(95%CI)**
**Age**				
19–35	34 (36.6)	59 (63.4)	1	1
36–48	43 (30.9)	96 (69.1)	1.2 (0.74, 2.29)	1.2 (0.64, 2.24)
>49	54 (53.5)	47 (46.5)	0.5 (0.28, 0.89)	0.66 (0.34, 1.26)
**Emotional support**				
Yes	127 (41.9)	176 (58.1)	1	1
No	3 (10.3)	26 (89.7)	6.25 (1.85, 21.11)	3.1 (0.85, 11.26)
**Patient-provider communication**				
Good	110 (53.6)	95 (46.4)	1	1
Poor	21 (16.4)	107 (83.6)	5.8 (3.42, 10.14)	4.5 (2.49, 8.12)^**^
**Financial support**				
Yes	91 (27.3)	43 (12.9)	1	1
No	40 (12.01)	28 (8.4)	2.32 (1.46, 3.68)	2.83 (1.63, 4.91)^**^
**Quintile status**				
Poor	91 (51.3)	87 (48.9)	1	1
Middle	13 (23.6)	42 (76.4)	3.4 (0.69, 6.72)	2.86 (1.33, 6.17)^**^
Rich	27 (27)	73 (73)	2.8 (1.7, 4.8)	2.84 (1.46, 5.46)^**^
**Physical exercise**				
Yes	37 (40.66)	54 (59.34)	1	1
No	94 (38.84)	148 (61.2)	1.19 (0.66, 1.76)	1.20 (0.64, 2.14)

** p < 0.01, AOR: Adjusted odds ratio, COR: crudes odds ratio.

**Table 4:** The result of logistic regression for depression after controlling the effect of confounding factors among patients with breast cancer.

**Characteristics**	**Depression**		**COR**	**AOR**
	**N (%)**		**(95%CI)**	**(95%CI)**
	**No**	**Yes**		
**Age**				
19–35	35 (37.6)	58 (62.3)	1	1
36–48	42 (30.2)	97 (69.8)	1.4 (0.80, 2.42)	1.25 (0.68, 2.31)
>49	61 (60.4)	40 (39.6)	0.39 (0.22, 0.71)	0.48 (0.25, 0.90)^*^
**Patient-family communication**				
Good	117 (45.4)	141 (54.7)	1	1
Poor	20 (27.1)	54 (72.9)	2.24 (1.27, 3.95)	1.01 (0.52, 1.96)
**Patient-provider communication**				
Good	109 (53.2)	96 (46.8)	1	1
Poor	29 (22.7)	99 (77.5)	3.88 (2.36, 6.36)	3.36 (1.87, 6.02)^**^
**Financial support**				
Yes	100 (52.4)	91 (47.6)	1	1
No	38 (26.8)	104 (73.3)	3.1 (1.88, 4.80)	3.36 (1.95, 5.79)^**^
**Quintile status**				
Poor	88 (49.4)	90 (50.6)	1	1
Middle	13 (23.6)	42 (76.4)	3.16 (1.58, 6.28)	3.09 (1.44, 6.65)^**^
Rich	37 (37)	63 (63)	1.66 (1.01, 2.75)	1.54 (0.85, 2.77)

* p < 0.05

** p < 0.01, AOR: Adjusted odds ratio, COR: crudes odds ratio.

Compared with patients aged 19–35 years, the odds of developing depression were 52% lower among patients aged >49 years (*AOR* = 0.48, 95% *CI*: 0.25, 0.90). Compared with those who had good patient-provider communication, the odds of developing depression were 3.36 times higher among those who had poor patient-provider communication (AOR = 3.36, 95% CI: 1.87, 6.02), for those who did not have financial support, the odds of developing depression were 3.36 times higher than those who had financial support (AOR = 3.36, 95% CI: 1.95, 5.79). Compared with the poor, the odds of developing depression were 3.09 times higher among patients in the middle wealth quintile (AOR = 3.09, 95% CI: 1.44, 6.65).

## Discussion

### Prevalence of anxiety and depression among patients with breast cancer

The prevalence of anxiety in this study was 60.66%. Our finding was much lower than the prevalence in the Ghanaian study which found anxiety to be 92.5% (28). The discrepancy might be attributed to the fact that most of the participants in the Ghanaian study were newly diagnosed patients with breast cancer, explaining the much higher prevalence of anxiety.

Approximately in line with our finding, a study done in London, United Kingdom, showed a 50% prevalence of anxiety among patients with breast cancer (29). In contrast with our finding, a study done in Iran among 150 patients with cancer reported anxiety to be 46% (30). Our finding was much higher than a study done in Malaysia among 205 patients with breast cancer where they found the prevalence of anxiety to be 31.7% (22). In addition, our study has a much higher prevalence than a study done in India, in which they found the prevalence of anxiety to be 37.0% (31). The large sample size we used in comparison with the studies carried out in Malaysia and India may be the potential reason for the higher prevalence in our study.

In this study, we found the prevalence of depression to be 58.56%. This was in agreement with a study done in Nigeria where the study showed the prevalence of depression to be 60.7% (32). Our finding was also slightly similar to that of a study conducted in the Federation of Bosnia and Herzegovina where they used the Hamilton Depression Assessment Scale (HDRS), which revealed that 40.3% of respondents had depression (33). The disagreement between this report and our finding could be due to the difference in the depression measurement scale, we used HADS, while the Bosnian study used HDRS. In addition, the majority of our participants were young, which is potentially linked with a higher degree of depression.

Our finding indicated a higher degree of depression in patients with breast cancer than a study done in Malaysia, where they conducted a survey on 141 patients with breast cancer and found the prevalence of depression to be 19.1% (16). Another prevalence study done in Greece on 152 patients with breast cancer also revealed the degree of depression to be 38.1% (34). The potential explanation for the observed disparity may be the variance in sample size between the current study and the two studies described above. On the other hand, the prevalence of depression in our study was much lower than that of a study in Ghana where the authors showed an 84.2% prevalence of depression (28). The higher prevalence in Ghana might be due to the late presentation of breast cancer cases in the country, and another possible explanation could be the patients' history of depression prior to the diagnosis of breast cancer which accounts for 33% of the total participants. In line with our finding, a study done in the United Kingdom reported almost 50% of patients with breast cancer have depression and anxiety or both (29). Our study had different findings when compared with the prevalence of a study done among patients with breast cancer in Ethiopia which reported the prevalence of depression to be 25% (19). The potential reason for this disparity might be: (1) the disparity in the instrument used to screen depression; they used PHQ-9, while we used HADS; and (2) the set up difference. They collected data from five private clinics (hospitals) as well as two government hospitals, and private sectors are known for good patient-provider interaction and better service delivery, which might have contributed to the lesser degree of depression observed in their study.

### Factors associated with anxiety and depression

In this study, patient-provider communication and financial support were significantly associated with both anxiety and depression. The odds of having depression and anxiety were found to be higher among those who had poor patient-provider communication. In line with our finding, a study done in Nigeria (32) and Croatia (33) stated that the compassionate relationship that the physician had with patients was very important in appeasing depression and anxiety symptoms. Furthermore, the way patients are being told about their treatment options and possible side effects would help reduce depressive symptoms in patients with breast cancer. More than providing information, however, the manner in which information is given is also very significant. The insensitive manner in which patients are frequently informed of their condition can contribute to increased anxiety and depression in patients with breast cancer (32).

In addition, financial support was another important predictor which was found to be significantly related to both anxiety and depression. The odds of having depression and anxiety were more likely to be higher among those who did not have financial support. Studies were in good agreement with our findings (31, 35). Studies showed that patients with breast cancer with good economic status were less likely to be anxious and depressed than their counterparts (28, 31, 36, 37). In contrast with the above findings, our study did not show the protective effect that good economic status had on depression and anxiety. The possible reason for this difference may be that in Ethiopia, cancer care is provided free of charge to all those with an insurance ID which is called “Tena medin” in the local language, Amharic. As a result, any patient, wealthy or poor, will have access to treatment: chemotherapy, surgery, radiotherapy, and hormonal therapy. In view of the fact that Tikur Anbessa Hospital is the only center in the country with radiotherapy, it is unavoidable for every patient, rich or poor, to visit the hospital for holistic treatment. Therefore, the better off might be more anxious about the thought of being able to afford cancer care but not being able to get holistic access as well as treatment despite having the means might cause anxiety among this group.

In our study, age was significantly associated with depression. Our finding showed that age was negatively associated with depression in which the odds of having depression were found to decrease as age increased. In the current study, younger patients were more likely to be depressed than the aged ones. In agreement with our findings, a study done in India showed that younger patients are more likely to have depression (31), and another study in Afro-American participants indicated that younger patients with cancer experienced depression than older ones (38). Other studies were also in good agreement with our findings (39, 40). The possible explanation for this correlation might be that young patients are less likely to expect to experience chronic illness. Furthermore, the fear of early death related to the thought of unsatisfied life goals among young patients might make them prone to depression. Another plausible explanation for this could be that younger patients tend to worry about losing their breast due to mastectomy. Given that breast is a symbol of femininity in various cultural entities, the absence of that body part is considered to affect attraction toward the opposite sex. As a result, the majority of younger patients in our study were frustrated that they cannot attract their sexual peers due to their condition and that they could face fertility issues due to the side effects of chemotherapy and mastectomy. Furthermore, patients in our study indicated that they appear to worry a lot when their hair falls off after the first chemotherapy cycle, as a result, they are forced to trim or shave their hair and wear hair coverings that are not routine for a young Ethiopian woman. This results in their surroundings raising concerns about their new appearance, and this resulted in stressful experiences for our participants.

In our study, none of the clinical characteristics showed statistical significance with depression and anxiety. In contrast with our finding, studies showed that clinical factors, such as stage of cancer, metastasis, and treatment type were significantly associated with anxiety and depression (32, 41, 42). The most plausible explanation for this could be that our established patients are not well informed about their condition. Patients may know what kind of disease they have, but they were not well informed about the grades, stages, and metastases. There are two plausible explanations for not informing the patient. One was that families were found to be overly defensive, not wanting patients to hear anything, especially in the case of elderly patients. Second, the patient flow at the Tikur Anbessa Hospital's Oncology Center was found to be extremely high, with one physician assessing 100–120 patients each day, making it extremely difficult to go into depth about each patient's health.

### Limitation

The study was a cross-sectional study. Moreover, it was conducted in the capital city of Ethiopia, Addis Ababa, which might direct us to the selection of better-offs and more educated ones, which in turn limits the generalizability of our findings. In addition, the tool we used to assess patient-provider, patient-family communication, and emotional support was not validated.

## Conclusion

In this study, the prevalence of anxiety and depression was high among patients with breast cancer, and the lack of financial support and poor patient-provider interactions were significant predictors of anxiety and depression among this group of patients. Patients with cancer need regular screening for mental health and better emotional support from their healthcare providers and families to prevent and treat anxiety and depression.

## Data availability statement

The raw data supporting the conclusions of this article will be made available by the authors, without undue reservation.

## Ethics statement

The studies involving human participants were reviewed and approved by Institutional Review Board of Addis Ababa University School of Public Health. The patients/participants provided their written informed consent to participate in this study.

## Author contributions

Conceptualization and investigation: WB and WH. Data curation: WB, WL, and WT. Formal analysis and writing the original draft: WB. Methodology: WB, WL, WT, WH, and MK. Project administration: WB, WL, and MK. Supervision: WB, MK, and WH. Validation: WB and WL. Writing, reviewing, and editing: WL, WT, MK, and WH. All authors contributed to the article and approved the submitted version.

## Conflict of interest

The authors declare that the research was conducted in the absence of any commercial or financial relationships that could be construed as a potential conflict of interest.

## Publisher's note

All claims expressed in this article are solely those of the authors and do not necessarily represent those of their affiliated organizations, or those of the publisher, the editors and the reviewers. Any product that may be evaluated in this article, or claim that may be made by its manufacturer, is not guaranteed or endorsed by the publisher.
